# S100A6: molecular function and biomarker role

**DOI:** 10.1186/s40364-023-00515-3

**Published:** 2023-09-05

**Authors:** Yidian Wang, Xuewen Kang, Xin Kang, Fengguang Yang

**Affiliations:** 1 Department of Joint Surgery, Honghui Hospital, Xi’an Jiaotong University, Xi’an, Shanxi China; 2 Department of Orthopedics, Lanzhou University Second Hospital, Lanzhou, China; 3 The Second Clinical Medical College, Lanzhou University, Lanzhou, China; 4 Orthopaedics Key Laboratory of Gansu Province, Lanzhou University Second Hospital, Lanzhou, China; 5 The Orthopedics Department of the Second Hospital of Lanzhou University, 82 Cuiying Men, Lanzhou, Gansu Province 730000 PR China

**Keywords:** S100A6, Biomarker, Cytoskeleton, Cellular stress response, Cell differentiation, Tumor, Stem cells

## Abstract

S100A6 (also called calcyclin) is a Ca^2+^-binding protein that belongs to the S100 protein family. S100A6 has many functions related to the cytoskeleton, cell stress, proliferation, and differentiation. S100A6 also has many interacting proteins that are distributed in the cytoplasm, nucleus, cell membrane, and outside the cell. Almost all these proteins interact with S100A6 in a Ca^2+^-dependent manner, and some also have specific motifs responsible for binding to S100A6. The expression of S100A6 is regulated by several transcription factors (such as c-Myc, P53, NF-κB, USF, Nrf2, etc.). The expression level depends on the specific cell type and the transcription factors activated in specific physical and chemical environments, and is also related to histone acetylation, DNA methylation, and other epigenetic modifications. The differential expression of S100A6 in various diseases, and at different stages of those diseases, makes it a good biomarker for differential diagnosis and prognosis evaluation, as well as a potential therapeutic target. In this review, we mainly focus on the S100A6 ligand and its transcriptional regulation, molecular function (cytoskeleton, cell stress, cell differentiation), and role as a biomarker in human disease and stem cells.

## Introduction

S100A6 (also called calcyclin) is a Ca^2+^-binding protein that belongs to the S100 protein family. Although S100A6 is a cytoplasmic protein, it can be located in the nuclear and cytoplasmic membranes in the presence of Ca^2+^ [1, 2]. One S100A6 monomer can bind to two Ca^2+^ ions with different affinities. One Ca^2+^ binds to the atypical EF-hand loop at the N-terminus of the molecule, and the other Ca^2+^ binds to the typical EF-hand loop at the C-terminus [3]. The binding of Ca^2+^ induces a conformational change in S100A6, exposes the hidden hydrophobic region, and induces interactions with the target protein, and Ca^2+^ signal transduction [4, 5]. So far, many S100A6-interacting proteins have been found, which are distributed in the cytoplasm (such as annexins II, VI, XI, CacyBP/SIP, and Sgt1) [3, 6–9], nucleus (such as lamin A/C, p53, and FOR20) [10–12], cell membranes (such as receptor for advanced glycation end products (RAGE), and integrin β) [13, 14], and extracellular space (such as lumican, and proline/arginine-rich end leucine-rich repeat protein (PRELP)) [15]. S100A6, combined with its ligand, plays an important role in biological processes, such as cytoskeletal function, stress response, cell proliferation, cell differentiation, and signal transduction. S100A6 can also bind to Zn^2+^; however, the binding site of Zn^2+^ is different from that of Ca^2+^ [16]. To date, no research has shown that S100A6 has potential Zn^2+^-dependent activity.

S100A6 exists in different mammalian cells and tissues, and its expression is substantially increased in fibroblasts and epithelial cells [17]. S100A6 is also expressed in neurons [18], myocardial cells [19], glial cells [20], lymphocytes [21], platelets [22], smooth muscle cells [23], and other cell types. Recent studies have shown that S100A6 is abnormally expressed in various tumor lesions, such as melanoma [24], lung [25], colorectal [26], pancreatic [27], and liver cancers [28]. It plays different regulatory roles in tumor invasion, cell proliferation, and migration, making it a potential therapeutic target. S100A6 is not only expressed in cells but also secreted out of cells [29], and can be detected in various body fluids, such as the extracellular matrix and blood [6]. Therefore, the differential expression of S100A6 in specific diseases makes it a useful biomarker for disease diagnosis and differentiation [30]. This has been one of the lucrative research directions for S100A6 in recent years.

In this review, we mainly focus on the S100A6 ligand and its transcriptional regulation, molecular function (cytoskeleton, cell stress, cell differentiation, etc.), and biomarker role in human diseases and stem cells.

## S100A6-interacting proteins

When S100A6 binds to Ca^2+^, it induces a conformational change resulting in the exposure of hydrophobic surfaces, which are now free to interact with some proteins [4, 5]. Therefore, S100A6 can interact with many proteins distributed in the nucleus, cytoplasm, membrane, and outside of the cell (Table 1), indicating that it has multiple molecular functions. Almost all the interactions with recognized S100A6-interacting proteins are Ca^2+^-dependent.

**Table 1:** Subcellular localization of S100A6 ligands

Cytoplasm	Nucleus	Cell membrane	Extracellular matrix
Annexins II, VI and XI, Tropomyosin, Caldesmon, Calponin, GAPDH, Lysozyme, CacyBP/SIP, Sgt1, Melusin, FKBP52, Cyp40, CHIP, PP5, FKBP38, Hop, Kinesin light chain, Tom70	Importin α, p53, p63, p73, lamin A/C, FOR20, FOP, OFD	RAGE, integrin β1	lumican, PRELP, IGFBP-1

*GAPDH* Glyceraldehyde-3-phosphate dehydrogenase, *Cyp40* Cyclophilin 40, *CHIP* C-terminus of Hsc70-interacting protein, *PP5* Protein phosphatase 5, *Hop* Hsp90/Hsp70-organizing protein, *Tom70* Translocase of outer mitochondrial membrane70

In the cytoplasm, the previously discovered S100A6-interacting proteins are annexin II, VI, and XI, tropomyosin, caldesmon, calponin, glyceraldehyde-3-phosphate dehydrogenase (GAPDH), and lysozyme [31–38]. In addition to the unknown functions of GAPDH and lysozyme interacting with S100A6 respectively, the interactions between these proteins and S100A6 in a Ca^2+^-dependent manner are closely related to the regulation of the actin cytoskeleton and membrane dynamics. CacyBP/SIP is an S100A6 ligand originally purified from Ehrlich ascites tumor cells [7], which was later confirmed to be expressed in all rat tissues at different levels [39]. The modular structure of CacyBP/SIP is important for bringing Siah-1 and Skp1 proteins into the polyubiquitination of β-catenin in the intact SCF-like complex consisting of Skp1, Culin and F-box protein [40]. Sgt1, a suppressor of the G2 allele of Skp1, which is a protein with a 20% homologous sequence to CacyBP/SIP, was originally found in yeast [41]. Therefore, it was speculated to interact with S100A6. This was confirmed by subsequent studies, which found that Sgt1 binds to S100A6, S100A1, S100A4, S100B, and S100P in a Ca^2+^-dependent manner [42]. Similar to CacyBP/SIP, Sgt1 is widely expressed in different tissues of rats, and its protein and mRNA levels in the brain, skeletal muscle, and spleen are higher than those in other tissues [43]. Some studies have suggested that β-catenin degradation is mediated by the SCF complex, which ubiquitinates the phosphorylated form of β-catenin [44]. Recent studies suggest that β-catenin levels depend on the CacyBP/SIP-Siah1 complex, which ubiquitinates non-phosphorylated β-catenin [9]. Sgt1, which is homologous to CacyBP/SIP, can ubiquitinate yeast proteins Cln1 and Sic1p. Therefore, Sgt1 is believed to affect the function of the ubiquitin ligase complex in mammalian cells [3]. When evaluating the general motif characteristics of S100A6-binding proteins, melusin was found to be similar to CacyBP/SIP and Sgt1, including the CS domain and flexible C-terminal region [3]. Melusin protects cardiac function in a mouse model of chronic hypertension [45]. In recent years, it has been reported that α- and β-tubulin can bind to S100A6 and may participate in the regulation of the cytoskeleton [29]. In addition, S100A6 can also bind to several other cytoplasmic ligand proteins that contain the TPR or CS domains, including FKBP52, cyclophilin 40 (Cyp40), C-terminus of Hsc70-interacting protein (CHIP), protein phosphatase 5) (PP5)FKBP38, Hsp90/Hsp70-organizing protein (Hop), kinesin light chain, and translocase of outer mitochondrial membrane70 (Tom70) [46–48].

Several S100A6-interacting proteins are present in the nucleus, such as importin α. Previous studies have shown that S100A6 specifically binds to the armadillo repeats of importin α in a Ca^2+^-dependent manner, resulting in the formation of importin α complexes in vivo and in vitro and inhibition of the nuclear localization signal (NLS). This suggests that S100A6 may regulate the nuclear transport of NLS-cargo in response to an increase in intracellular Ca^2+^ concentration [49]. Three p53 family members, namely p53, p63, and p73, are S100A6-interacting proteins; additionally, S100A6 may regulate their transcriptional activity [11, 50]. Lamin A/C, a protein known to be involved in colon cancer [10], interacts with S100A6, which also provides indirect evidence that S100A6 is involved in colon cancer. Recent studies have shown that three centrosomal proteins, FOR20, FOP, and OFD, which are related to cilia formation, also interact with S100A6 in a Ca^2+^-dependent manner [12].

RAGE and integrin β1 are S100A6-interacting proteins located on the cell membrane. Although S100B and S100A6 have similar structures, they interact with different RAGE extracellular domains. The experimental data showed that S100B and S100A6 have different regulatory effects on cell survival. At micromolar concentrations, S100B promoted cell proliferation, whereas, at the same concentration, S100A6 triggered cell apoptosis. Although the two S100 proteins induce ROS formation, S100B recruits PI3K/AKT and nuclear factor-κB (NF-κB), while S100A6 activates JNK. More importantly, S100B and S100A6 regulate cell survival in a RAGE-dependent manner. S100B specifically interacts with the V and C1 domains of RAGE, while S100A6 specifically interacts with the C1 and C2 domains of RAGE [51]. Another study also showed that the S100A6-binding site is located in the C1 domain of RAGE [52]. This study showed that S100A6 adopts a dimer conformation that is distinct from all known S100 dimers. The combination with S100A6 also induced a unique dimer conformation of RAGE, which seemed to be suitable for signal transduction and recruitment of intracellular effectors [52]. However, some studies indicate that S100A6 interacts with the V domain of RAGE [53]. Further studies are needed to clarify the discrepancy. Similar to other S100A6-interacting proteins, integrin β1 interacts with S100A6 in a Ca^2+^-dependent manner. The application of an integrin β1-specific antibody reverses the effect of S100A6 on cell adhesion and proliferation [14], which may be due to the blockade of S100A6-integrin interaction. S100A6 protein activates integrin-linked kinase (ILK), focal adhesion kinase (FAK), and p21-activated kinase (PAK) [29].

Three S100A6-interacting proteins, lumican, PRELP, and insulin-like growth factor binding protein 1 (IGFBP-1), were found in the extracellular matrix of Wharton’s jelly in healthy and preeclampsia patients. Lumican and PRELP exist in both healthy patients and preeclampsia patients, while IGFBP-1 only exists in the tissues of preeclampsia patients [15] and may be associated with the development of preeclampsia. The interaction between S100A6 and these proteins is direct, with IGF-1 competing with S100A6 for binding to IGFBP-1 [15].

## Regulation of S100A6 transcription

**Fig. 1:**
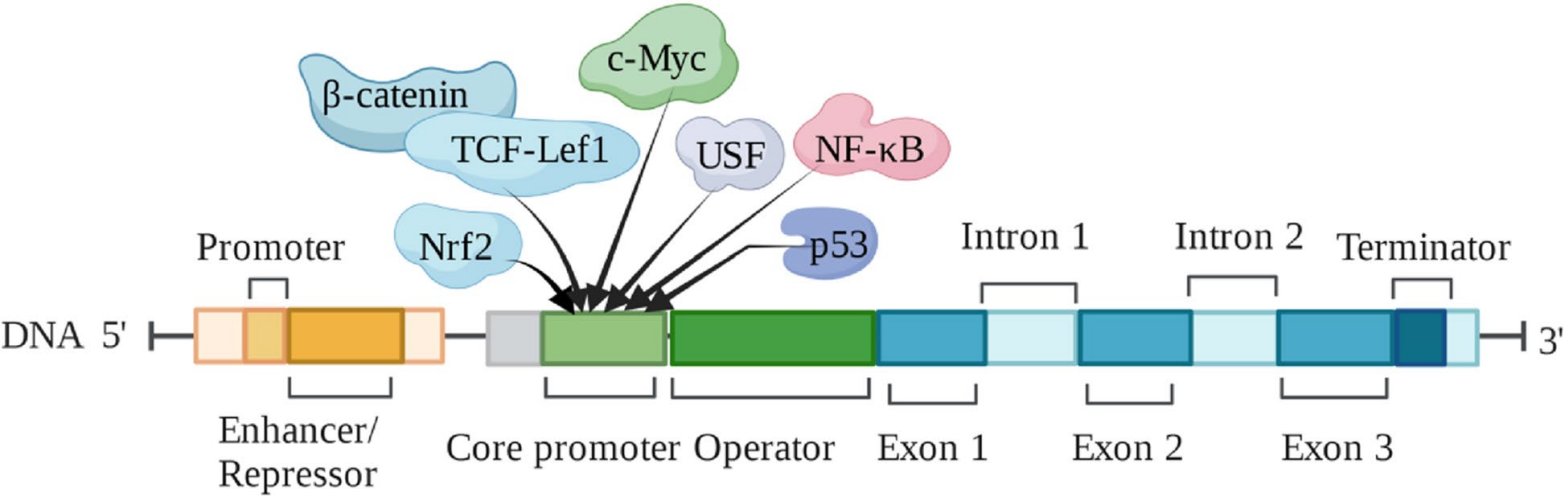
S100A6 gene structure and transcription factors (based on literature reports)

Published data have revealed several transcription factors that regulate S100A6 expression (Fig. 1). Dysfunction of skin keratinocyte differentiation in diabetes is closely related to the impairment of skin barrier function. c-Myc expression is increased in human immortal keratinocytes (HaCaT) cultured in high glucose, diabetic rats, and wound marginal keratinocytes in diabetes patients. High sugar levels, by activating the WNT/β-catenin pathway, increased the expression of c-Myc and inhibited the differentiation of HaCaT cells, while c-Myc combined with the S100A6 promoter directly regulated the expression of S100A6. Inhibition of c-Myc transcriptional activity can alleviate the differentiation dysfunction caused by hyperglycemia or c-Myc overexpression [54]. This suggests that c-Myc and S100A6 are potential therapeutic targets. In the non-metastatic human colorectal cancer cell line SW480, S100A6 was found to be activated by co-transfection of β-catenin and TCF-Lef1 transcription factors [10]. The TCF/LEF family transcription factor domain contains the N-terminal β-catenin-binding domain, which mediates the binding of TCF to β-catenin. Therefore, β-catenin and TCF-Lef1 might activate S100A6 expression after binding.

P53 is a key regulator of apoptosis and the cell cycle, and S100A6 is believed to be involved in cell-cycle control. Transcriptional regulation experiments in HeLa cells revealed that p53 inhibited the S100A6 promoter in a dose-dependent manner. Inadequate inhibition of this gene by p53 mutants may be responsible for the overexpression of S100A6 and the cell-cycle dysregulation observed in cancer tissues [55]. NF-κB (p65 subunit) strongly induced promoter activity of the porcine S100A6 gene, while the NF-κB inhibitor pyrrolidine dithiocarbamate hampered this activity. In addition, promoters containing mutant NF-κB-binding sites were considerably less active than those containing the wild-type [56].

In addition, the upstream stimulatory factor (USF) can not only binds to the E-box sequence of -283/-278 bits of the S100A6 promoter sequence but also the second E-box motif of -593/-588 bits. The USF1/USF2 heterodimer is the main dimeric form that induces S100A6 transcription [57, 58]. In the oxidative stress model, it was found that the antioxidant response element (ARE) located at the -290/-281 bases of the S100A6 gene promoter overlaps with the E-box sequence recognized by USF, and the mutation of ARE led to the inhibition of Ca^2+^-stimulated S100A6 promoter activity. In addition to binding to USF, another protein complex, shown by competitive electrophoretic mobility shift assay (EMSA), can bind to ARE. DNA affinity chromatography and western blotting revealed that the Nrf2 transcription factor could bind to the fixed S100A6 gene promoter segment. This indicates that oxidative stress inducers activate S100A6 through the ARE sequence in the promoter [59]. In addition to the regulation by these transcription factors, S100A6 expression is affected by epigenetic modifications such as histone acetylation and DNA methylation [60, 61]. More research is needed to reveal the regulatory mechanism of S100A6 expression.

## S100A6 and cytoskeleton

Several studies have shown that some S100 protein family members participate in the regulation of cytoskeleton assembly and degradation [62, 63]. S100A6 is one of the most studied members. Inhibition of S100A6 expression in rat pulmonary fibroblasts weakens cell proliferation, accompanied by flat cell morphology and microfilament destruction marked by tropomyosin [64]; this not only shows that S100A6 is related to the cytoskeleton but also promotes cell proliferation. The S100A6 binding partner cofilin-1 has been found in mouse lung fibroblasts (NIH3T3 cell line) [65]. S100A6 binds to cofilin-1 in a Ca^2+^-dependent manner, increasing the affinity of cofilin-1 for F-actin and reducing actin filament breakage induced by cofilin-1. In addition, S100A6 stabilizes fibers by inhibiting fiber depolymerization in the presence of cofilin-1. In conclusion, S100A6 regulates actin filament dynamics by controlling the activity of cofilin-1 in a Ca^2+^-dependent manner [65]. Additionally, S100A6 can directly interact with G-actin and F-actin and preferentially interact with a subtype of tropomyosin (Tpm1.8). S100A6 and tropomyosin bind to the same fiber group and the presence of tropomyosin on the microfilament promotes the binding of S100A6. S100A6 forms complexes with actin and tropomyosin in NIH3T3 cells. These results indicate that S100A6 may regulate the tissue and functional properties of microfilaments through direct interactions with actin and tropomyosin [66].

Osteosarcoma has a high degree of malignancy. Most patients have metastatic or micrometastatic lesions, and metastatic disease is detected in less than 15% when they have clinical manifestations [67]. Some studies have shown that S100A6 is overexpressed in human osteosarcoma. Inhibition of S100A6 expression can inhibit cell adhesion and promote cell migration and invasion. In contrast, S100A6 overexpression can enhance cell adhesion and inhibit cell invasion [68]. The influence of S100A6 on the phenotypes of osteosarcoma cells, such as adhesion, migration, and invasion, may be inextricably related to its cytoskeletal regulation.

Whether actin is present or not, S100A6 will only cross-link with tropomyosin chains in the presence of Ca^2+^, and the combination of tropomyosin and actin will not lead to the dissociation of tropomyosin [34]. S100 has been shown to regulate the assembly of brain tubulin in vitro and to act on microtubule nucleation and elongation through Ca^2+^ mediation. This process is also affected by temperature, Ca^2+^, pH, and in other physical and chemical environments [62]. In addition, myeloid-related protein (MRP)14 (S100A9) and MRP8 (S100A8) interact with the cytoskeleton to perform related functions [69–71]. Many members of the S100 family are involved in the regulation of cytoskeleton-related structures and functions. Perhaps the domain shared by the family members is combined with specific cytoskeletal sites, playing a specific role in different time sequences and jointly completing the mission. When evaluating the impact of S100A6 on tumor invasion, migration, and other functions related to the cell bone ring, research on regulating physical and chemical environmental conditions is also valuable and may identify potential therapeutic targets.

## S100A6 and cellular stress response

The cellular stress response is a defensive or adaptive response of cells to endogenous or exogenous injury factors. When cells are stimulated by physical and chemical factors, a series of signal transductions occur internally to promote the rapid expression of stress-related molecules and play a role in promoting cell apoptosis or survival. S100A6 participates in the cell stress response, and its expression level increases under oxidative stress [59], hypertension [72], ischemia [73], mechanical stimulation [74], radiation [75], and other stresses.

The Ca^2+^-dependent interaction with other proteins is critical for S100A6 to perform biological functions. Chaperones are particularly important in cellular stress, especially Hsp90 and Hsp70. Hsp90 and Hsp70 perform the most basic physiological functions in cells, such as protein folding, stretching, transport, oligomer formation, and depolymerization, to maintain cell survival and function. Under adverse conditions such as stress, they can improve the resistance of cells and play a role in stress protection [76, 77]. Hsp90 and Hsp70 are closely bound to chaperone proteins when performing their functions. The chaperoning process is initiated by the interaction between Hsp70 and the client protein, and then the newly formed complex is transferred to the Hsp90-co-chaperone to form a more complex compound (Hsp70-client protein-Hsp90-co-chaperone) called a fold [4].

**Fig. 2:**
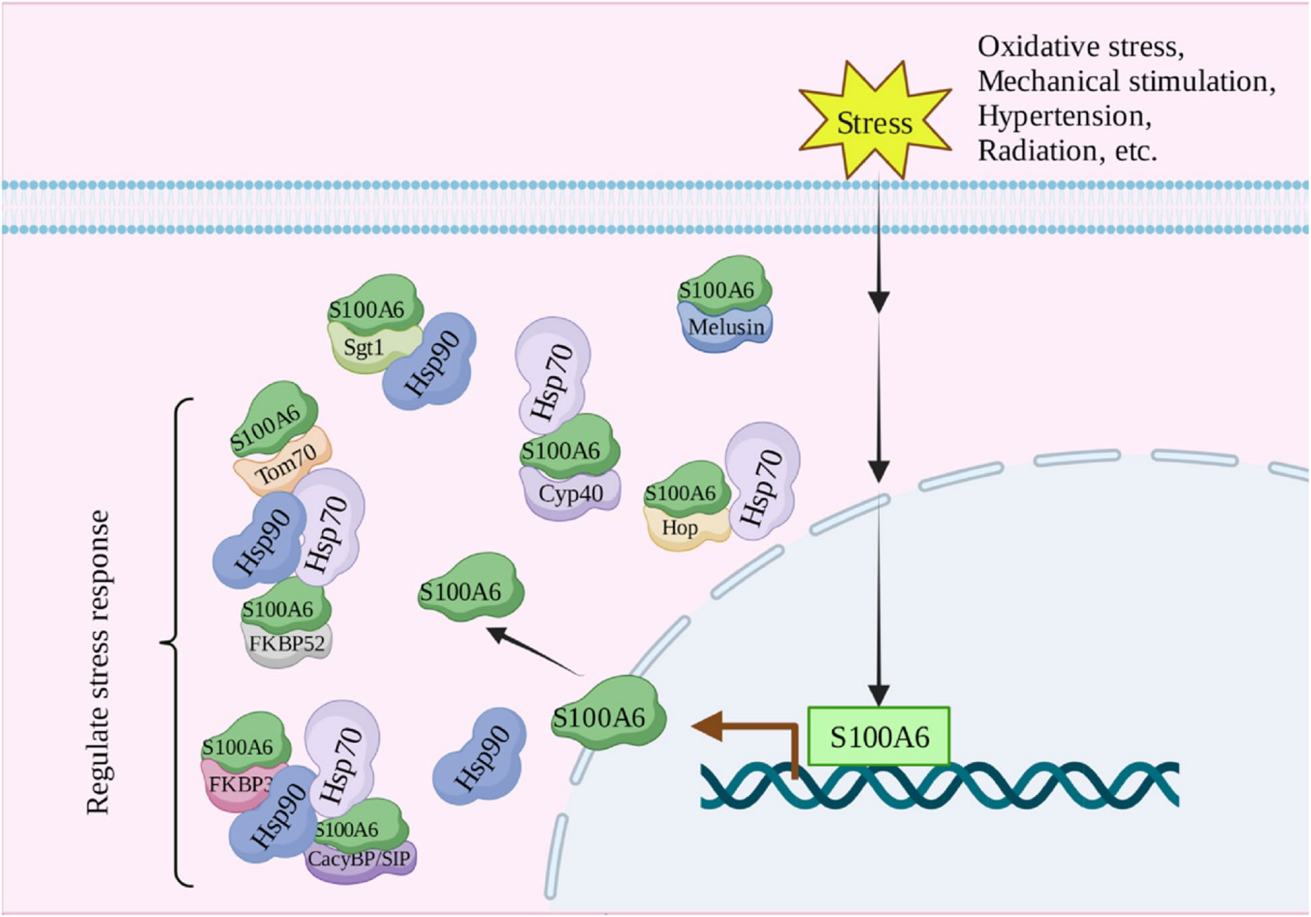
S100A6 is involved in the regulation of cellular stress response. Cyp40, cyclophilin 40; Hop, Hsp90/Hsp70-organizing protein; Tom70, translocase of outer mitochondrial membrane70

S100A6 can interact with many of the common partners of Hsp70/ Hsp90, such as kinesin light chain), Hop, Cyp40, Tom70, FKBP52, CHIP, and melusin [3, 7, 42, 46, 48, 78, 79]. This is because their interaction with S100A6 involves the same domain, namely the TPR domain or the CS and/or SGS domain [46, 48, 78, 80]. Although it is clear that S100A6 participates in the cell stress response (Fig. 2), most of the results are from related studies, and the in-depth mechanism needs to be ascertained further.

## S100A6 and cell differentiation

The immense diversity of S100 family proteins proves that under various physiological and pathological conditions of cells in the human body, S100 family proteins have multiple biological correlations with cell growth, differentiation, and survival [63]. S100B, a member of the S100 family, is an EF-hand type calcium-binding protein that is widely expressed in astrocytes and participates in the regulation of various intracellular activities, including proliferation and differentiation [81]. Using small interfering RNA technology to reduce the expression level of S100B in the astrocytoma cell line GL15 and the Müller cell line MIO-M1 led to the rapid decomposition of stress fibers; additionally, the collapse and migration of F-actin on the plasma membrane were reduced, and a star shape was acquired. In addition, silencing S100B in GL15 and MIO-M1 cells resulted in more glial fibrillary acidic protein filaments, which marked astrocyte differentiation. These effects depend on S100B interacting with Src kinase to stimulate the PI3K/Akt and PI3K/RhoA pathways [81]. These results suggest that S100B may help reduce the differentiation potential of astrocytes.

As a member of the S100 family, S100A6 has also been found to participate in cell differentiation. Some studies, using an in vitro epidermal differentiation model and stable S100A6 knockout or overexpression HaCaT cells (human immortalized keratinocytes), found that S100A6 mRNA levels decreased significantly during primary keratinocyte differentiation, and that the change in S100A6 expression was not related to DNA methylation but might be affected by epileptogenic factors (ΔNp63 transcription factor and retinoic acid). In conclusion, this study showed that an increase in S100A6 content in keratinocytes significantly changes the speed and degree of epidermal differentiation [82]. HaCaT cells were cultured with high glucose or with keratinocytes from diabetic rats and patients with diabetes, and the expression of S100A6 increased [54]. This occurs because high sugar levels activate the Wnt/β-catenin pathway, which increases the expression of c-Myc, and c-Myc can directly regulate the expression of S100A6 by combining with the S100A6 promoter. The dysfunction of HaCaT cell differentiation caused by overexpression of c-Myc or high glucose can be prevented by knocking out S100A6. These findings suggest that c-Myc upregulation by high glucose inhibits HaCaT differentiation by directly activating S100A6 transcription. Therefore, c-Myc and S100A6 are potential targets for the treatment of chronic diabetic wounds [54].

**Fig. 3:**
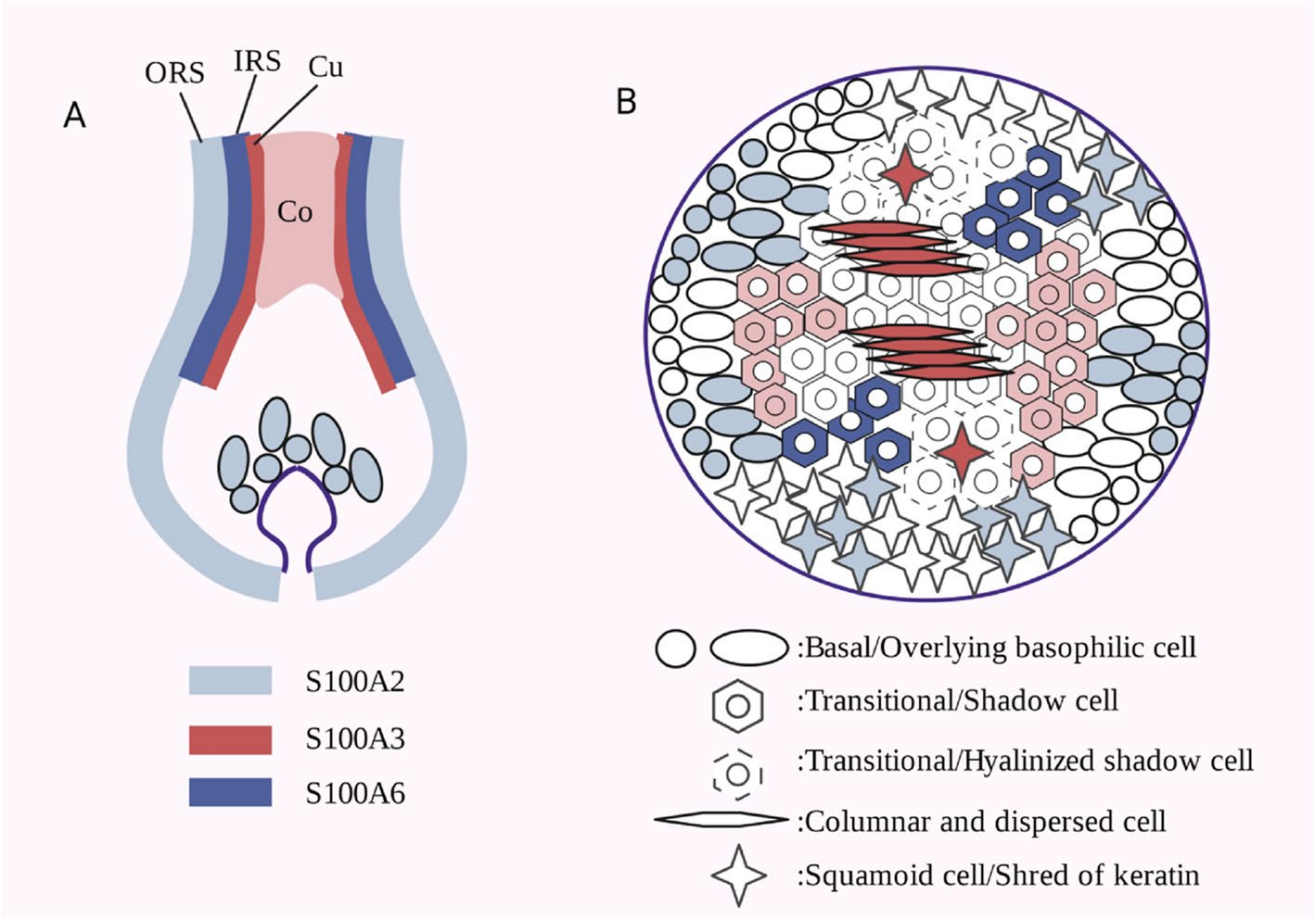
Schematic localization of S100A2, S100A3 and S100A6. **A** Hair matrix cells (some S100A2 positive) proliferated and differentiated into cortical and cuticular cells (S100A3 positive), and the inner root sheath cells (S100A6 positive). Outer root sheath cells (S100A2 positive) surround the hair bulb. Co, cortex of hair; Cu, cuticle of hair; IRS, inner root sheath; ORS, outer root sheath. **B** Tumour island of pilomatrixoma. Basophil cells (patellar S100A2 positive) divide into adhesion shadow cells (S100A3 positive), transparent shadow cells (S100A6 positive), and possibly columnar or dispersed cuticle cells (S100A3 positive). The outer root sheath cells are inherited by squamous cells (S100A2 positive). (Adapted from reference 83)

S100A6 and its family members also participate in the regulation of tumor cell differentiation. Pilomatrixomas are common benign skin tumors containing differentiated hair matrix cells. Although some S100 proteins are expressed in hair follicles in a tissue-specific manner (for example, S100A2 is in the outer root sheath, S100A3 is in the cortex and stratum corneum, and S100A6 is in the inner root sheath), little is known regarding their distribution in trichoblastoma tissues with abnormal differentiation. Immunohistochemistry and double immunofluorescence microscopy were used to detect squamous cells stained with anti-S100A2 antibody, putative root sheath cells, and basophilic and potential hair stromal cells that were occasionally stained. S100A3 staining can be seen in both transitional cells and putative cortical cells and hair-like structures of scattered and surrounding cells (putative keratinocytes). Anti-S100A6 antibody marks some S100A3 negative transitional cell chains, which may be inner root sheath cells [83]. This shows that hair stromal tumors retain a certain degree of differentiation with different hair-forming cells (Fig. 3) [83]. However, the co-expression of S100A6 and MMP9 is related to the progression of skin squamous cell carcinoma [84] and may be involved in some characteristic differentiation of squamous cell carcinoma cells, which needs to be proven. S100A6 is expressed in human osteosarcoma cell lines. Knockdown of S100A6 can improve the osteogenic differentiation activity of mesenchymal stem cells, whereas overexpression of S100A6 inhibits osteogenic differentiation. bone morphogenetic protein 9-induced bone formation is enhanced by S100A6 inhibition. This suggests that S100A6 inhibits the osteogenic differentiation of osteosarcoma [85]. Moreover, S100A6 may participate in thymocyte differentiation [86]. Few studies have examined the relationship between S100A6 expression and cell differentiation. Therefore, knowledge regarding this mechanism is lacking and needs to be complemented with future research. Cell differentiation and proliferation are closely related, whereas S100A6 and cell proliferation are often related to tumor diseases. Therefore, we will introduce S100A6 in combination with cell proliferation and tumor diseases.

## Biomarker role of S100A6

### S100A6 as a cancer biomarker

S100A6 is expressed in various tumor diseases, and variations in its expression in different diseases and at different stages of disease development make it a biomarker for disease diagnosis, differentiation, and prognosis evaluation. Accurate diagnosis of thyroid tumors is challenging, and S100A6 plays a potential auxiliary role in the differentiation between follicular thyroid tumors and papillary thyroid carcinomas [87]. The combination (through the decision tree) of the S100 protein family (including S100A6), galectin-3, and its ligand profile can establish an improved identification standard for benign and atypical meningiomas [88]. The unique staining pattern in S100A6 immunohistochemistry may be helpful for the identification of pilar leiomyomas (LM), angeleiomyomas (ALM), and cutaneous leiomyosarcomas (LMS) that are difficult to identify. S100A6 was expressed in some skin smooth muscle tumors and the difference in staining intensity between LM and LMS was statistically significant. A strong S100A6 staining is conducive to the diagnosis of LMS, whereas negative staining supports the diagnosis of LM [89]. Moreover, S100A6 mRNA was significantly elevated in cholangiocarcinoma (CCA), while no significant increase was observed in hepatocellular carcinoma (HCC). The detection of S100A6 mRNA and protein expression levels in tissue samples by northern blotting and immunohistochemical staining may be another useful marker for differentiating between CCA and HCC [90].

S100A6 is closely associated with the prognosis and malignancy of many tumor diseases. The expression of S100A6 in lung squamous cell carcinoma is significantly correlated with patient age and tumor differentiation. S100A6 overexpression is closely related to the poor prognosis of lung squamous cell carcinoma and is considered an independent poor prognostic factor for lung squamous cell carcinoma [91]. In addition, S100A6 is related positively to the invasion of lung adenocarcinoma, especially bronchial adenocarcinoma (BAC). The current results showed that S100A6 immunohistochemistry can be used as a marker to evaluate the malignancy of adenocarcinoma with BAC components [92]. S100A6 and S100A2 are also associated with the progression of thyroid tumors [93]. Although the expression level of S100A6 is significantly increased in human and mouse CCA tumor samples, the serum level of S100A6 is not changed in patients with CCA; therefore, it is not suitable for the diagnosis of CCA. However, compared with patients with low S100A6 levels, CCA patients with elevated S100A6 levels show a trend of impaired prognosis, which supports its use as a biomarker for CCA prognosis [94]. S100A6 may also be a prognostic marker and potential therapeutic target for patients with intrahepatic cholangiocarcinoma (ICC) [95]. MALDI-IMS proteomics has shown that S100A6, S100A10, and thioredoxin can be used for risk stratification of the metastatic potential of papillary thyroid carcinoma [96].

S100A6 not only plays a role in the differential diagnosis, prognosis, and malignant degree prediction of tumor diseases but is also a diagnostic and monitoring indicator of some tumor diseases. Current data show that S100A6 levels increase hierarchically during the occurrence of pancreatic cancer. Measuring the content of S100A6 in pancreatic juice may help identify early pancreatic cancer or high-risk individuals who may develop pancreatic cancer [97]. Moreover, some studies have shown that S100A6 mRNA quantification is a promising diagnostic method and therapeutic target for pancreatic cancer [98]. Serum S100A6 expression is a potential and effective marker for bladder cancer detection. Applying this serum marker to clinical practice can lead to less invasive examinations for patients and help identify life-threatening cancers earlier than current methods [99]. Through proteomic analysis of tumor interstitial fluid, S100A6 was found to be a potential risk marker for screening cholangiocarcinoma [100]; however, it was difficult to obtain test samples. The detection of S100A6 expression levels in gastric cancer tissue and S100A6 detection in serum showed that it has diagnostic and prognostic value and is likely to become a potential therapeutic target [101, 102]. In non–small-cell lung cancer (NSCLC), S100A2 and S100A6 are significantly increased in the early stage and may have potential as biomarkers [103]. S100A6 is also expressed in ovarian and other cancer tissues. The serum concentration of S100A6 was found to be related to the experimental tumor load and clinical disease stage. These data suggest that S100A6 may be useful in detecting and/or monitoring ovarian cancer when used in combination with other biomarkers [104]. In contrast to the increased expression in other tumors, loss of S100A6 protein expression is very common in the development of prostate cancer, which may occur at an early stage. S100A6 expression loss may be a useful diagnostic method for prostate cancer [105].

The expression level of S100A6 in tumors can be used as a biomarker for differentiation, diagnosis, malignancy, and prognosis evaluation (Table 2). However, the detection of S100A6 in tumors must consider whether sample acquisition is simple and feasible and whether serological detection is more feasible. Most of the research results need to be verified by a large cohort and multicenter research. S100A6 is expected to bring about novel changes in the diagnosis and treatment of tumor diseases.

**Table 2:** S100A6’s biomarker role in tumor diseases

**Disease**	**Specimen**	**Sample size**	**Function**	**References**
Thyroid Tumors	Tissue	Papillary carcinomas 10; follicular carcinomas 9; follicular adenomas 10	Discrimination between follicular thyroid tumors and papillary thyroid carcinoma	[87]
Meningiomas	Tissue	Benign meningiomas 39; atypical meningiomas 24	Discriminatory between the benign and the atypical meningiomas	[88]
Cutaneous smooth muscle neoplasms	Tissue	Pilar leiomyoma 22; angioleiomyoma 22; cutaneous leiomyosarcoma 22	Discriminatory between pilar leiomyoma, angioleiomyoma, and cutaneous leiomyosarcoma	[89]
Cholangiocarcinoma	Tissue	Cholangiocarcinoma 18; hepatocellular carcinoma 20	Differentiation cholangiocarcinoma from hepatocellular carcinoma	[90]
Lung squamous cell cancer	Tissue	Lung squamous cell cancer 177	Independent unfavorable prognostic factor for lung squamous cell cancer	[91]
Pulmonary adenocarcinoma	Tissue	Lung adenocarcinomas formalin-fixed and paraffinembedded 92; and frozen surgical specimens 6	Estimation of malignancy in adenocarcinoma	[92]
Thyroid carcinomas	Tissue	Thyroid neoplasms 141	Involved in the progression of papillary carcinoma	[93]
Cholangiocarcinom	Serum	Cholangiocarcinom 112; healthy controls 42	Biomarkers of poor prognosis	[94]
Cholangiocarcinoma	Tissue	/	Prognostic marker and potential therapeutic target	[95]
Thyroid carcinoma	Tissue	Thyroid carcinoma 118	Risk stratification regarding metastatic potential in papillary thyroid carcinoma	[96]
Pancreatic cancer	Pancreatic Juice	Pancreatic Juice 98	Early diagnosis and identification of high precancerous lesions	[97]
Pancreatic Cancer	Pancreatic juice and tissue	Pancreatic carcinoma 33; normal tissue samples 39	Diagnostic marker and therapeutic target	[98]
Bladder cancer	Serum	Bladder cancer 50; healthy controls 30	Early diagnostic marker	[99]
Cholangiocarcinoma	Tumor interstitial fluid	Cholangiocarcinoma 3; paired samples 3	Potential risk marker for screening of cholangiocarcinoma	[100]
Gastric cancer	Serum	Gastric cancer 103; healthy controls 72	Prognostic biomarker	[101]
Gastric cancer	Tissue	Gastric cancer 1; paired sample 1	Diagnostic marker	[102]
Non-small cell lung cancer	Serum	Non-small cell lung cancer 141; healthy controls 150	Diagnostic marker	[103]
Epithelial ovarian cancer	Serum	**/**	Detecting and/or monitoring ovarian cancer	[104]
Prostate cancer	Tissue	Benign 66; premalignant 10; malignant 66; metastatic prostate 5	Diagnostic marker	[105]

### Biomarker role of S100A6 in stem cells

Stem cells exist in various tissues of the body, and their location is called a niche. Stem cells can proliferate and differentiate into offspring of some special cell types and are the cell source needed for tissue maintenance and regeneration. Owing to its potential to repair tissue damage, it is the focus of tissue engineering and other regenerative repair research. However, stem cells are scarce and lack universal markers, making their isolation and identification difficult [30]. In recent years, the development of various omics technologies has helped to promote stem cell-related research. Increasing evidence shows that S100A6 is expressed in various stem cells and has the potential to become a biomarker.

Epidermal stem cells (ESCs) mainly exist in the basal layer of the interfollicular epidermis (IFE) and hair follicle bulge area under the sebaceous gland [106]. Epidermal cells, also known as keratinocytes, serve as barriers against direct contact with the external environment, shedding and renewing periodically. S100A6 protein and mRNA have been found to exist in the mouse hair follicle bulge area and outer root sheath (adjacent to the bulge) [107]. Similarly, microarray analysis showed that fluorescence-activated cell sorting (FACS) based on fluorescence labeling enriched S100A6 gene expression in label-retaining cells (LRC) isolated from the skin of transgenic mice [108]. Wnt-10b can also promote skin-derived CD34 and CD49f double-positive cells (enriched epithelial stem cells or progenitor cells) to express classical markers of bulge stem cells, including S100A6, Lhx2, Keratin15, Sox9, and NFATc1 [109]. With the development of single-cell sequencing technology, evidence of S100A6 expression in mouse hair follicle bulge cells and the basal layer of the hair follicle inner epidermis is more abundant [110, 111]. These findings have led researchers to list S100A6 as a marker for epidermal stem cells.

Mesenchymal stem cells (MSCs) exist in the connective tissue and organ stroma, with the bone marrow being the most abundant reservoir. Because the bone marrow is the main source of MSCs, they are also called bone marrow mesenchymal stem cells. Mesenchymal stem cells can express specific molecules and differentiate into osteoblasts, adipocytes, and chondrocytes in vitro [112]. Single-cell sequencing (scRNA-seq) has shown that S100A6 expression is upregulated in human synovial fluid-derived mesenchymal stem cells (hSF MSCs) [113]. Additionally, published literature shows that S100A6 can be secreted by Wharton’s jelly mesenchymal stem cells and is associated with integrin β1 binding to activate ILK, FAK, and PAK [29]. This suggests that S100A6 may be a marker of mesenchymal stem cells.

According to current knowledge, there are mainly two regions of neural stem cells in the brain of adult mice: the subventricular zone (SVZ) near the lateral ventricles of the forebrain, and the subgranular layer of the dentate gyrus of the hippocampal formation [30]. Some studies have shown that S100A6 is highly expressed in neural stem cells labeled with the sex-determining region Y-box 2, brain lipid-binding protein, and glial fibrillary acidic protein by immunofluorescence multiple labeling. It was also shown for the first time that S100A6 is a specific marker of neural stem cells and astrocyte precursors and may also be particularly important for the generation of astrocytes in the adult hippocampus [114]. It has also been found that most of the sox2-positive cells located in the dentate gyrus (DG) of mice express S100A6 at the same time [115]. In addition, S100A6 immunostaining is higher in the subependymal zone (SEZ) but lower in the medial subependymal zone (MEZ) [116].

The surfaces and cavities of internal organs are covered by epithelial cells. The intestinal epithelium has strong regenerative and fast renewal abilities. The base of the intestinal crypt is considered the niche of intestinal stem cells (ISCs) and the source of new intestinal epithelial cells. However, owing to the rapid renewal of the intestinal epithelium, the proliferation of cells in the crypt has always been active, and its real reserve intestinal stem cells have proven difficult to establish [117]. Through pedigree tracking, it was found that Lgr5-expressing cell derivatives contain radiation-resistant ISCs, which are crucial for the regeneration of irradiated intestinal epithelium [118]. S100A6 was found to be expressed in mouse resting intestinal stem cells that survived whole-body irradiation and were able to reconstruct the intestinal epithelium [118].

The “cancer stem cell hypothesis” has two main concepts: one is that tumors originate from tissue stem cells or their direct progeny cells because the self-renewal process that is strictly regulated under normal circumstances is out of balance; second, the tumor contains a kind of cell sub-component, which retains the key stem cell characteristics. These characteristics include self-renewal, which drives tumorigenesis and abnormal differentiation, leading to cellular heterogeneity [119]. Although this hypothesis has been put forward for more than 150 years, it has not received increasing attention in the field of cancer research until progress in cancer stem cell biology has been made in recent years. As a common abnormally expressed gene in tumors, S100A6 has been found in the lateral population of cancer stem cells (CSCs) isolated from brain tumors of transgenic mice forming glioma mouse models [120]. Gene expression analysis showed a 31.5-fold increase in S100A6 gene expression (and a 14.7-fold increase in S100A4 gene expression) in these cells compared with most tumor cells. Immunofluorescence staining confirmed the presence of S100A6 and S100A4 proteins in the cancer subpopulation of primary human glioma, and their abundance was correlated with tumor grade [120]. Some studies have isolated and identified breast CSCs through tumor ball culture, identified the changes in protein expression in CSCs compared with non-CSCs using LC–MS/MS, and identified a group of differential genes (PTMA, S100A4, TNXRD1, COX-1, COX-2, KRT14, and FTH1), representing possible molecular targets [121]. CSCs exhibit self-renewal, pluripotency, tumor initiation after transplantation, and resistance to radiotherapy or chemotherapy drugs, which may be the cause of cancer recurrence. Therefore, research on CSCs is of great significance to improve the understanding and diagnosis of cancer.

Hematopoietic stem cells (HSCs) located in the bone marrow can differentiate into various lineages of blood cells [122]. After continuous differentiation, HSCs enter the multipotent progenitor (MPPs) stage and then differentiate into precursors of two blood cell lineages: common lymphoid progenitors (CLP) or common myeloid progenitors (CMP). After a series of differentiations, the former becomes T and B lymphocytes or natural killer cells (NK), while the latter becomes monocytes, granulocytes, red blood cells, and megakaryocytes [122]. However, S100A6 knockout HSCs reduced the total number of HSC in the HSC chamber, reduced the output of the myeloid system, and increased the number of apoptotic HSCs in the stable state [123]. This indicates that S100A6-knockout HSCs have impaired self-renewal and regeneration capacities. Transcriptional and proteomic analyses have shown that S100A6 is a key HSC regulator. Activation of the Akt pathway is the main mechanism of S100A6 promoting HSCs self-renewal via regulation of mitochondrial metabolic function and Hsp90 protein quality [123].

Immunohistochemistry, RT-PCR, and scRNA-seq techniques revealed the expression of S100A6 in epidermal, neural, cancer, hematopoietic, and epithelial stem cells (Table 3). These data indicate that S100A6 is closely related to cellular stemness. However, there are few studies on S100A6 as a marker for stem cell identification and characterization, and more research is needed.

**Table 3:** S100A6’s biomarker role in stem cells

**Name of Stem cells**	**Location**	**Function**	**References**
Epidermal stem cells	Base of the interfollicular epidermis; bulge part of a hair follicle	Regeneration of hair follicle and part of epidermis	[107–111]
Mesenchymal stem cells	Human synovial fluid; Wharton’s jelly	Differentiate into osteoblasts, chondrocytes, etc	[29, 113]
Neural stem cells	Subgranular zone, subventricular zone	Differentiation of neurons, astrocytes and oligodendrocytes	[114–116]
Intestinal stem Cells	Intestine	Intestinal epithelial regeneration	[117, 118]
Cancer stem cells	In various tumors	Tumor formation, recurrence, drug resistance, etc	[119–121]
Hematopoietic stem cells	Bone marrow	Any lineage that differentiates into blood cells	[123]

### Biomarker role of S100A6 in other diseases

S100A6 is expressed in tumor diseases and stem cells and has a potential biomarker role. S100A6 has similar functions in other diseases. Serum calumin, S100A6, and cytokinin 2 have been confirmed as biomarkers in patients with systemic sclerosis with skin involvement, and S100A6 is related to the number of active digital ulcers [124]. Another study used a functional proteomic approach to analyze bronchoalveolar lavage samples from patients with pulmonary fibrosis systemic sclerosis (PF-SSc), smokers, and nonsmokers. The MetaCore network of this study suggests the use of C3a, APOAI, 14–3-3e, SPFA2, and S100A6 as potential biomarkers. These upstream molecules are involved in pulmonary fibrosis, innate immunity, and vascular injury in PF-SSc patients [125]. In addition, in a transgenic mouse model of amyotrophic lateral sclerosis (ALS), it was found that reactive astrocytes (not motor neurons) highly expressed S100A6 in several regions (including 12 pairs of nerve roots) in the brain stem, while S100A6 was negative in the sublingual nucleus. However, in the dorsal root, the reactive astrocytes from the white matter and the anterior horn were highly reactive [126]. Therefore, the overexpression of S100A6 is considered a valuable diagnostic marker for ALS [126]. Specific expression of S100A6 in many diseases results in its rich functional characteristics. Revealing its function in specific diseases is of great significance for understanding and diagnosing related diseases.

## Conclusions

S100A6 belongs to the S100 protein family, and is a calcium-binding protein. S100A6 has numerous molecular functions related to the cytoskeleton, cell stress, cell proliferation, and cell differentiation. S100A6 also has many interacting proteins that are distributed in the cytoplasm, nucleus, cell membrane, and outside the cell. Almost all these proteins interact with S100A6 in a Ca^2+^-dependent manner, and some also have a specific motif, which is responsible for binding to S100A6 to initiate biological effects. The expression of S100A6 is regulated by several transcription factors (such as c-Myc, P53, NF-κB, USF, Nrf2, etc.). The level of S100A6 expression depends on the specific cell type and activated transcription factors in specific physical and chemical environments, and is also related to epigenetic modifications, such as histone acetylation and DNA methylation.

S100A6 can be used as a biomarker for diagnosis, differential diagnosis, and prognosis evaluation because of its differential expression in various diseases and at different stages of the same disease. In tumor diseases, it is also related to the degree of malignancy and can be used to monitor related diseases and as a potential therapeutic target. S100A6 can be used not only as a biomarker of disease but also as a biomarker of stem cells. Stem cells are the source of cells needed for tissue maintenance and regeneration, and have the potential to repair tissue damage. They are the research focus in tissue engineering and other regeneration and repair fields. However, stem cells are scarce and lack universal markers, making their isolation and identification difficult. With the development of various omics technologies, increasing evidence has shown that S100A6 is expressed in various stem cells and is a promising biomarker that may aid in carrying out stem cell-related work.

S100A6 is a key molecule in the body and is involved in many pathophysiological processes. Its differential expression in normal tissues or diseases, especially in various diseases or at different stages of the same disease, gives it the functional characteristics of biomarkers. It also has the potential to be a target for the treatment of some diseases. The increasing interest in S100A6 may help improve the understanding of these diseases and advance diagnostic and treatment methods.

## Data Availability

Not applicable.

## Abbreviations

RAGE: Receptor for advanced glycation end products
PRELP: Proline/arginine-rich end leucine-rich repeat protein
GAPDH: Glyceraldehyde-3-phosphate dehydrogenase
Cyp40: Cyclophilin 40
CHIP: C-terminus of Hsc70-interacting protein
PP5: Protein phosphatase 5
Hop: Hsp90/Hsp70-organizing protein
Tom70: Translocase of outer mitochondrial membrane70
NLS: Nuclear localization signal
IGFBP-1: Insulin-like growth factor binding protein 1
ARE: Antioxidant response element
EMSA: Electrophoretic mobility shift assay
LM: Leiomyomas
ALM: Angeleiomyomas
LMS: Leiomyosarcomas
CCA: Cholangiocarcinoma
HCC: Hepatocellular carcinoma
BAC: Bronchial adenocarcinoma
ICC: Intr ahepatic cholangiocarcinoma
NSCLC: Non–small-cell lung cancer
ESCs: Epidermal stem cells
IFE: Interfollicular epidermis
FACS: Fluorescence-activated cell sorting
LRC: Label-retaining cells
MSCs: Mesenchymal stem cells
scRNA-seq: Single-cell sequencing
hSF MSCs: Human synovial fluid-derived mesenchymal stem cells
SVZ: Subventricular zone
DG: Dentate gyrus
MEZ: Medial subependymal zone
ISCs: Intestinal stem cells
CSCs: Cancer stem cells
HSCs: Hematopoietic stem cells
MPPs: Multipotent progenitor stage
CLP: Common lymphoid progenitors
CMP: Common myeloid progenitors
NK: Natural killer
